# The Differential Effect of Antibodies on Radiographic Progression in Rheumatoid Arthritis

**DOI:** 10.31138/mjr.31.4.393

**Published:** 2020-12-22

**Authors:** Amal Minocha, Sebi Kukran, Philip Yee, Muhammad Nisar

**Affiliations:** 1 University College London Medical School, Bloomsbury, London, United Kingdom; 2 Rheumatology Department, Luton & Dunstable University Hospital NHSFT, Luton, United Kingdom

**Keywords:** Erosions, RA, radiographic, antibodies

## Abstract

**Background/Objectives:**

The presence of bony erosions in patients with RA is a marker of disease severity and once present they are largely irreversible. Previous studies have shown that the presence of both rheumatoid factor (RF) and anti-cyclic citrullinated peptide (ACPA) antibodies is associated with erosive burden. The aim of our study is to determine the strength of relationship between antibody status and the presence of radiographic erosions at diagnosis.

**Methods:**

A retrospective study of patients diagnosed with RA at a large university teaching hospital between January 1981 and December 2018. Clinical records were reviewed to determine antibody status, diagnosis date, duration of symptoms, DAS-28, age, ethnicity and whether the 1987 RA criteria was met. The presence of erosions at diagnosis were determined from plain film radiographs reports of hands and feet of patients. Statistical analysis was done using a Chi Square Model and Mann Whitney two-tailed U test.

**Results:**

There were 774 patients diagnosed with RA in our cohort. 367 (47%) of them were RF+/ACPA+, 87 (11%) were RF+/ACPA-, 66 (9%) were RF-/ACPA+ and 254 (33%) were antibody negative. 127 patients had erosions at the time of diagnosis. Patients in the double positive group had a significantly higher (p=0.003) erosion burden compared to the double negative group i.e. 21.5% in RF+/ACPA+ versus 11.0% in RF-/ACPA- group. The erosion burdens in RF+/ACPA- and RF-/ACPA+ groups were 13.7% and 12.1% respectively.

**Conclusions:**

Our results show that patients RF+/ACPA+ have nearly two-fold higher incidence of radiographic erosions than patients who are RF-/ACPA-.

## INTRODUCTION

Rheumatoid arthritis (RA) is a chronic inflammatory disease which can have devastating outcomes if left untreated.^1^ Chronic synovial inflammation is the hallmark of RA. This inflammation can in turn cause resorption and inadequate bone formation which can be visualised on plain radiographs as erosions. Over time, these erosions and bone loss can lead to total joint destruction. Erosions are associated with negative patient outcomes including disability, financial burden and disease severity.^2,3^ Once present, they are largely irreversible.^4^ Modern treatments have allowed RA to evolve from an inexorably progressive disease to one in which we can induce remission. It is imperative that we are able to identify patient with erosive disease at presentation so that focused treatment can be initiated early with a view to prevent radiographic progression and lessen the impact of this destructive disease.

The 1987 American College of Rheumatology (ACR) criteria for RA focussed on persisting symptoms but failed to identify patients with early inflammatory arthritis.^5^ Studies have shown that erosions can develop as early as 8 weeks from the beginning of symptoms,^6^ and there is therefore a window of opportunity to act. Thus, many rheumatology departments see patients with suspected RA urgently through ‘early inflammatory arthritis’ pathway to ensure prompt specialist review.^7,8^ Despite recognition of the need for prompt referral, some patients unfortunately have erosions at presentation which is strongly correlated with radiographic progression and poorer outcomes.^9^ Hence, it is of paramount importance to improve early detection and instigation of aggressive therapy in patients with poor prognostic factors.

The best-known antibodies associated with RA are anti-cyclic citrullinated peptide (ACPA) and rheumatoid factor (RF). ACPA has a higher specificity for the diagnosis of RA compared to RF, with the combination of both resulting in an even higher specificity.^10^ In healthy individuals, the presence of both antibodies has been shown to predict the development of RA, with ACPA having the highest predictive value.^11^ In patients with existing RA, the presence of both antibodies is associated with increased disease severity in terms of functional disability, the absence of remission, and presence of erosions.^12^ Studies have shown that RA patients who are RF positive have more erosions than those who are RF negative, and that RF itself is an independent risk factor for developing bone erosions.^13,14^ Likewise, ACPA is associated with increased bone loss and faster erosive changes in patients with RA.^15^ The aim of our study was to determine the relationship of antibody status with erosive disease and whether being double +ve confers higher risk than single positive antibody status.

## MATERIALS AND METHODS

A retrospective study was undertaken of all 1015 patients diagnosed with rheumatoid arthritis at a large district general hospital during the period of January 1981 to December 2018. The hospital has a total of 641 beds across all disciplines and serves a local population of over 400,000 with about 41% of the population from cultural and ethnic minority backgrounds; the main groups being Asian and African-Caribbean.

AM and SK reviewed patient clinical records electronically of every patient diagnosed with RA to determine: RF and ACPA antibody status, date of diagnosis, duration of symptoms, DAS-28 score, age, ethnicity, and whether the diagnosis met the 1987 Rheumatoid Arthritis criteria. ACPA antibodies were obtained at any point during the patient’s management until December 2018. The presence of radiographic erosions at diagnosis were also determined from analysing the reports of plain film radiographs of the hands and feet of all patients. These radiographs were independently reported by consultant radiologists who had no prior knowledge of the antibody status of the patients. When this was unclear or there were discrepancies in the data, PY reviewed and reported the radiographs. The project was approved on 7 Sep 2018 (approval number 11/2018-19/Medicine/Rheumatology)

## Statistical Analysis

Statistical analysis was conducted using IBM SPSS Statistics 23 software and Epi Info version 7.0 (CDC Atlanta USA). Chi square model was utilised to ascertain if there was a significant relationship among the four groups. Mann Whitney two-tailed U test was employed to determine the significance of relationship between the double negative group and other arms for all variables including disease duration and delta change in DAS28. Significance level was predefined at 0.05. COX regression analysis and multivariate analysis were also performed to identify factors influencing erosive disease.

## RESULTS

### Patients’ demographics

A total of 1015 patients were diagnosed with RA based on clinical judgement during the time period. Of these, a total of 774 patients had available results for both antibodies (at some point in their disease course) and radiographs present at diagnosis, and thus were included in the study. 240 (31%) patients were male and 534 (69%) were female, with an age range of 17 to 90 years for the cohort. 543 (70%) patients identified their ethnicity as White, 179 (23%) as Asian, 37 (5%) as Afro-Caribbean, and 15 (2%) as Other. The duration of symptoms ranged for the patient cohort from 0.5 to 250 months with a median of 6 months while the duration of disease ranged from 4 to 455 months with a median of 49 months. DAS 28 scores for the patient cohort ranged from 1.19 to 8.4 with a median of 4.4. Of the patient cohort, 367 (47%) were positive for both RF and ACPAs, 87 (11%) were positive for RF alone, 66 (9%) were positive for ACPA alone, and 254 (33%) were antibody negative. 449 (58%) patients were found to have their diagnosis meet the 1987 RA classification. The double positive subgroup had a median DAS28 score of 4.52 with 69% of patients in the subgroup meeting the 1987 criteria, whereas the double negative group had a median DAS 28 score of 4.13 with 43% of patients meeting the 1987 criteria. Patient demographics and antibody subset data is summarised in **Table 1**.

**Table 1.** Comparison of antibody status, duration of disease and symptoms across gender, age and ethnicity.

**Group**			**RF+/ACPA+**	**RF+/ACPA-**	**RF-/ACPA+**	**RF-/ACPA-**	**Total**
N (total)			367	87	66	254	774
Male, N			124 (34%)	26 (30%)	24 (36%)	66 (36%)	240 (31%)
Female, N			243 (66%)	61 (70%)	42 (64%)	188 (74%)	534 (69%)
Age range (years)			17–86	20–84	29–79	21–90	17–90
Duration of symptoms (m)		*Min.*	0.5	1	1	1	0.5
*Max.*		156	120	84	240	240	
*Median*		6	6	7	6	6	
Duration of disease (m)		*Min*	5	6	7	4	4
*Max*		455	271	307	420	455	
*Median*		56	59	60	35	49	
DAS 28		*Min.*	1.19	1.19	1.64	1.19	1.19
*Max*		8.4	7.23	7.42	7.81	8.4	
*Median*		4.52	4.38	4.10	4.13	4.4	
1987 +, N			255 (69%)	53 (61%)	32 (48%)	109 (43%)	449 (58%)
Ethnicities	*White*		262	65	36	180	543 (70%)
	*Asian*		85	16	23	55	179 (23%)
	*Afro-Caribbean*		18	3	4	12	37 (5 %)
	*Other*		2	3	3	7	15 (2 %)

### Additive effect of RF and ACPA on erosive disease

**Figure 1.**
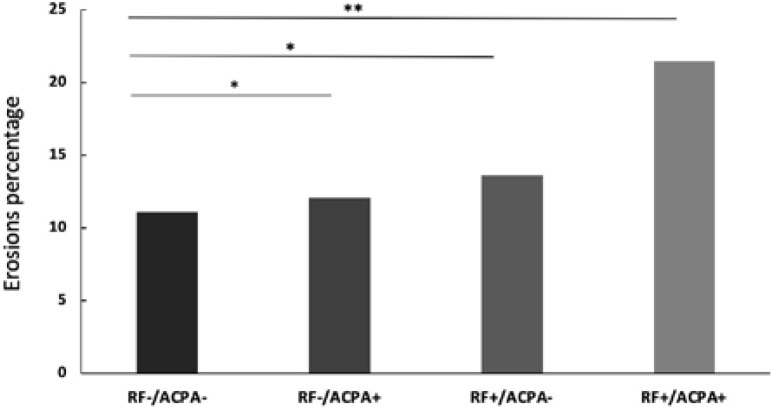


We looked at the reports of plain film radiographs for the presence of erosions in the hands or feet of every patient in the study. A total of 127 patients had erosions at the time of diagnosis. 79 of them were double positive for RF and ACPA, 12 were positive only for RF, 8 were positive only for ACPA and 28 were double negative. This is summarised in **Table 2.** Patients in the double positive group had a significantly higher (p=0.003) erosion burden compared to the double negative group, ie, 21.5% in RF+/ACPA+ compared to 11.0% in the RF-/ACPA- group. The erosion burdens in RF+/ACPA- and RF-/ACPA+ groups were 13.7% and 12.1% respectively. Mann-Whitney U test confirmed significance of the relation (p<0.05) for only double +ve group compared to double –ve (**Figure 1**).

**Table 2.** Comparison of the erosive burden between varying antibody status cohorts.

**Ethnicities**	**RF+/ ACPA+**	**RF+/ ACPA-**	**RF-/ ACPA+**	**RF-/ ACPA-**	**Total**	**Erosions in group, N**	**Erosions in group %**
White	262	65	36	180	543	97	17.9
Asian	85	16	23	55	179	18	10.1
Afro-Caribbean	18	3	4	12	37	9	24.3
Other	2	3	3	7	15	3	20

RF: rheumatoid factor; ACPA: anti-cyclic citrullinated peptide.

### Other factors influencing erosive disease

**Figure 2.**
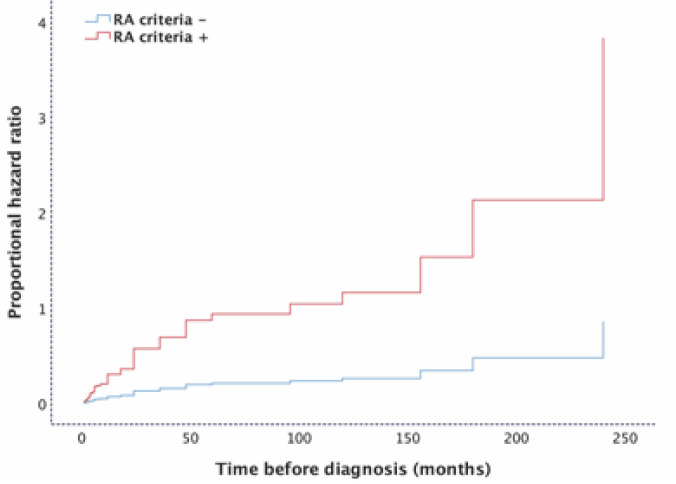


Analysis of the ethnicities within the population revealed that 24.3% of Afro-Caribbean patients, 17.9% of White patients, 10.1% of Asian patients, and 20% of patients identified as “Other” had erosions. This is summarised in **Table 3.** Other factors were also analysed when comparing the populations of erosive and non-erosive disease. The median DAS28 score was found to be 4.79 in the erosive population and 4.32 in the non-erosive population. There was a higher proportion of men (38%) in the erosive population compared to the non-erosive population (28%). The age range was found to be comparable in both groups. The percentage of patients meeting the 1987 RA classification criteria was, as expected, higher in the erosive group (83%) compared to the non-erosive group (60%) (**Table 4**). None of these relationships were statistically significant. COX regression analysis however showed that patients who met the 1987 criteria had more erosive disease compared to patients that had not met the criteria (β=0.223, 95% CI, 0.119 to 0.420, p<0.0001) (**Figure 2**).

**Table 3.** Comparison of antibody status and erosive disease amongst different ethnic groups

	**RF+/ACPA+**	**RF+/ACPA-**	**RF-/ACPA+**	**RF-/ACPA-**	**Total**
Total in group	367	87	66	254	774
Erosions +	79	12	8	28	127
% with Erosions	21.5	13.7	12.1	11.0	16.4

RF- rheumatoid factor, ACPA- anti-cyclic citrullinated peptide

**Table 4.** Comparison of DAS 28, scores gender, age, duration of symptoms and whether patients met 1987 RA criteria between population of patients with erosive disease and non-erosive disease.

**Erosive disease**			**Non-Erosive disease**	
DAS 28	*Min*	1.19		1.19
	*Max*	8.2		8.4
	*Median*	4.79		4.32
Gender (%)	*Female*	62		72
	*Male*	38		28
Age range		17 to 85		19 to 90
RA 1987 criteria+ (%)		83		60
Duration of symptoms	*Min*	1		0.5
	*Max*	240		240
	*Median*	9		6

In univariate analysis, age, gender, RF, ACPA, 1987 RA criteria and time before diagnosis predicted the erosive disease (p<0.05 for all), whereas DAS 28 and ethnicity were not significant (p>0.05). In multivariate analysis, gender 1.912 (1.005 to 3.009, p=0.02), time before diagnosis 1.011 (0.002 to 1.020, p=0.01), RA criteria 3.426 (1.680 to 6.933, p=0.001), and ACPA 2.186 (1.050 to 4.552, p=0.03), predicted erosive disease (**Table 5**).

**Table 5.** Factors predicting erosive disease in univariate and multivariate analysis

**Variable**	**Univariate predictors**	**P value**	**Multivariate predictors**	**P value**
	**odds ratio (95% CI)**		**Odds ratio (95% CI)**	
Age	1.009 (1.001 to 1.016)	**0.01**	1.015 (1.002 to 1.020)	0.11
Gender	1.807 (1.149 to 2.844)	**0.01**	1.912 (1.005 to 3.009)	**0.02**
Ethnicity	0.823 (0.589 to 1.149)	0.25		
RF	2.487 (1.504 to 3.117)	**0.001**	0.947 (0.458 to 1.954)	0.82
ACPA	2.262 (1.344 to 3809)	**0.001**	2.186 (1.050 to 4.552)	**0.03**
DAS 28	1.179(0.994 to 1.397)	0.58		
1987 RA criteria	4.060 (2.618 to 8.779)	**<0.001**	3.426 (1.680 to 6.933)	**0.001**
Time before diagnosis	1.007 (1.001 to 1.012)	**0.011**	1.011 (0.002 to 1.020)	**0.01**

RF: rheumatoid factor, ACPA: anti-cyclic citrullinated peptide

## DISCUSSION

Our study demonstrates the radiographic erosive burden in the RA cohort with respect to RF/ACPA status. Our results show that RF+/ACPA+ patients have significantly higher incidence of radiographic erosions than the RF-/ACPA- group, almost twofold. Patients with single antibody positivity have a trend towards higher erosive burden compared to the double antibody negative group; however, it is the combination of two antibodies which is strongly associated with erosive disease. Disease severity, although associated with erosive disease, was not significantly different in our cohort. Our study also shows that Afro-Caribbean group had higher erosive burden however numbers are small. However, in univariate analysis, ethnicity was not significantly associated with erosive disease.

The pathophysiology of bone erosive formation in rheumatoid arthritis involves the production of proinflammatory cytokines, receptor activator of nuclear factor kappa-B ligand (RANKL), synovitis and autoantibodies.^2^ It has been shown that ACPA is able to recognise citrullinated vimentin expressed on the surface of osteoclast precursor cells and the binding of the ACPA to the cell surface is able to increase cellular differentiation to bone-resorbing osteoclasts.^16^ Although the role rheumatoid factor plays in the pathogenesis of erosive disease has not been well defined in literature, there is evidence to suggest that there is a synergetic role of ACPA and RA in the pathogenesis of RA. The presence of RF-IgM or RF-IgA complexes in the presence of ACPA immune complexes has been shown to boost the Fc-gamma receptor mediated immune response, increasing the capacity of ACPA immune complexes to activate the complement cascade.^17^

This interaction between the autoantibodies and increased inflammatory process is further supported by a 2014 cohort study published by Sokolove et al.^18^ They grouped 1488 US veterans with RA according to their antibody status (ACPA+/RF+, ACPA-/RF+, ACPA+/RF-, ACPA-/RF-) and compared the levels of disease activity and serum levels of cytokines in each group. They found that the double positive subgroup exhibited higher levels of disease activity, higher C-reactive protein, and inflammatory cytokine levels than the double negative and single positive subgroups.^18^

Studies to date have come to differing conclusions with regards to the presence of single or double antibody status and higher erosive burden. Van Steenbergen et al. examined this association using two large European RA cohorts: the Leiden Early Arthritis Clinic (EAC) cohort of 678 patients with RA from Netherlands, and the Better Anti-Rheumatic PharmacOTherapy (BARFOT) cohort of 715 Swedish patients with early RA. Within the EAC cohort, it was found that all three seropositive subgroups of antibodies (ACPA+/RF+, ACPA-/RF+, ACPA+.RF-) had higher progressive erosive rates as shown on plain film radiographs compared to the double negative subgroup. Similar erosion scores between the ACPA+/RF+ group and the ACPA+/RF- group suggested RF did not give an additive effect on bone erosion in ACPA-positive patients. In ACPA-negative patients however, the presence of RF was associated with more severe erosive disease. These trends were also observed within the BARFOT cohort.^19^ Hecht et al. (2015) reached differing conclusions. They looked at 242 RA patients with respect to erosion number and size on high-resolution peripheral quantitative CT (HR-pQCT) scans of the metacarpophalangeal joints.^20^ They showed ACPA and RF to have an additive effect on erosion number and size, and interestingly, that RF influenced erosion size only in APCA-positive but not ACPA-negative patients. In our study, only the RF+/ACPA+ group was significantly associated with incidence of erosions compared with RF+/ACPA-, RF-/ACPA+ and RF-/ACPA- groups, reinforcing a synergistic effect of RF and ACPA pathways on each other in the role of bone erosion formation.

Currently, there is limited evidence regarding the effect of ACPA and RF in a real world clinical setting, ie, on plain film radiographic erosions. This is especially important given its associations with poorer patient outcomes. Plain films initially have more direct clinical relevance than more advanced imaging techniques, as they are commonly requested at baseline for a new patient with suspected inflammatory arthritis. Plain films are far more accessible and cost effective in modern health care settings. Moreover, the clinical relevance of erosions on advanced imaging such as MRI is unclear as ‘physiological’ erosions can be present in healthy controls and patients with other arthritides. For instance, one study has shown that only one in four MRI erosions progresses to an x-ray erosion over one year.^21^ Other studies have also supported the notion of overlap between erosions found on MRI between controls and RA patients.^22^ Additional factors with the potential to influence radiographic erosive burden, such as duration of disease, ethnicity, and relation to disease activity (DAS28), have also not previously been extensively explored in a quantitative fashion. This has provided the basis for our study. To our knowledge, this is the first study looking at the prognostic value of radiographs, antibodies and disease activity scoring in combination.

Our study has a reasonably large cohort which increases the validity of our results. The data set has also allowed us to explore potential differences between the erosive burden suffered by different ethnic groups and also genders. The radiographs used were easily accessible and mostly analysed by consultant radiologists who were unaware of the patients’ antibody status to reduce bias. Additionally, standardised scoring for disease severity such as the DAS 28 was used rather than objective assessments or comments made by clinicians in the patients’ notes. However, it is worth noting that this was a single-centre retrospective study, and involved cross-sectional evaluation at the point of diagnosis rather than long term follow-up and evaluation of new patients. Further observational studies are needed to determine the true relationship between double antibody positive patients and erosive burden.

## CONCLUSION

We have shown that patients RF+/ACPA+ have a significantly higher erosive burden compared to RF-/ACPA-, almost twofold.

**Key points:**
- Patients positive for both RF and ACPA have a significantly higher erosive burden at diagnosis compared to patients negative for both antibodies, as seen on plain film radiographs.
- Patients positive for only RF or ACPA also have a higher erosive burden at diagnosis compared to patients negative for both antibodies.
